# Supplementary material for: Design, Synthesis and Evaluation of New Multifunctional Benzothiazoles as Photoprotective, Antioxidant and Antiproliferative Agents
Source: Molecules. 2022 Dec 29;28(1):287. doi: 10.3390/molecules28010287 (PMC9822352; doi:10.3390/molecules28010287)
Supplement: Supplementary file 1 [file molecules-28-00287-s001.zip › molecules-2101179-supplementary.pdf]

DESIGN, SYNTHESIS AND EVALUATION OF NEW MULTIFUNCTIONAL BENZOTHIAZOLES AS PHOTOPROTECTIVE, ANTIOXIDANT AND ANTIPROLIFERATIVE AGENTS

Riccardo Barbari[1], Chiara Tupini[2], Elisa Durini[1], Eleonora Gallerani[3], Francesco Nicoli[3], Ilaria Lampronti[2], Anna Baldisserotto[1,*], Stefano Manfredini[1]

[1] Department of Life Sciences and Biotechnology, Section of Medicines and Health Products, University of Ferrara, Via Fossato di Mortara 17-19, I-44121 Ferrara, Italy

[2] Department of Life Sciences and Biotechnology, Section of Biochemistry and Molecular Biology, University of Ferrara, Via Fossato di Mortara 74, I-44121 Ferrara, Italy

[3] Department of Chemical, Pharmaceutical and Agricultural Sciences, University of Ferrara, via Luigi Borsari 46, I-44121 Ferrara, Italy

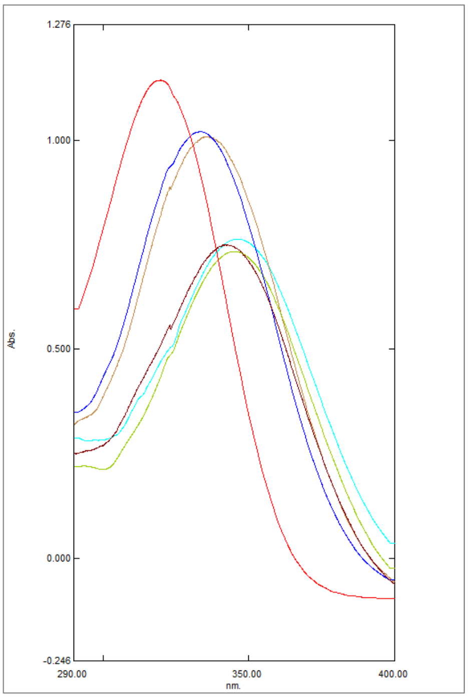

Figure S1. UV spectra of compounds **BZTidr1-6**: **BZTidr1** (red), **BZTidr2** (ocher), **BZTidr3** (blue), **BZTidr4** (light blue), **BZTidr5** (brown), **BZTidr6** (acid green).

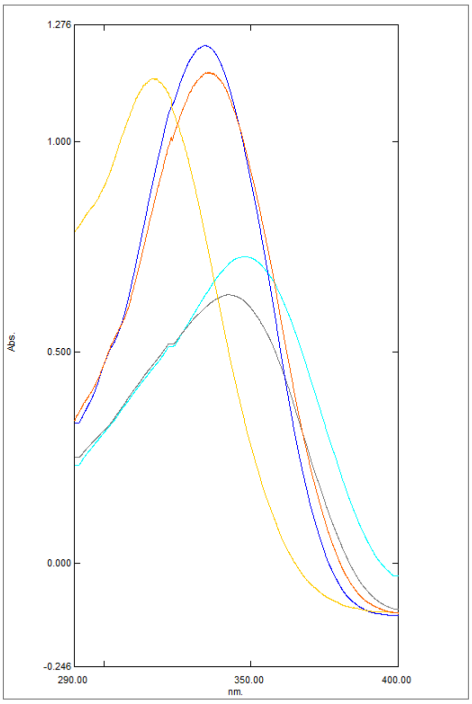

Figure S2. UV spectra of derivatives **BZTcin1-5**: **BZTcin1** (yellow), **BZTcin2** (orange), **BZTcin3** (blue), **BZTcin4** (light blue), **BZTcin5** (grey).

Table S1. Lambda max and ε values of the synthesized derivatives.

Compound	λmax (nm)	ε
**PBSA**	302.0	23881.86
**BZTidr1**	319.4	33223.32
**BZTidr2**	335.3	28637.64
**BZTidr3**	333.1	29641.85
**BZTidr4**	346.2	22183.99
**BZTidr5**	342.4	23462.08
**BZTidr6**	344.8	22071.63
**BZTcin1**	317.0	33033.71
**BZTcin2**	335.6	32240.17
**BZTcin3**	334.5	36271.07
**BZTcin4**	347.7	22387.64
**BZTcin5**	342.0	20688.20

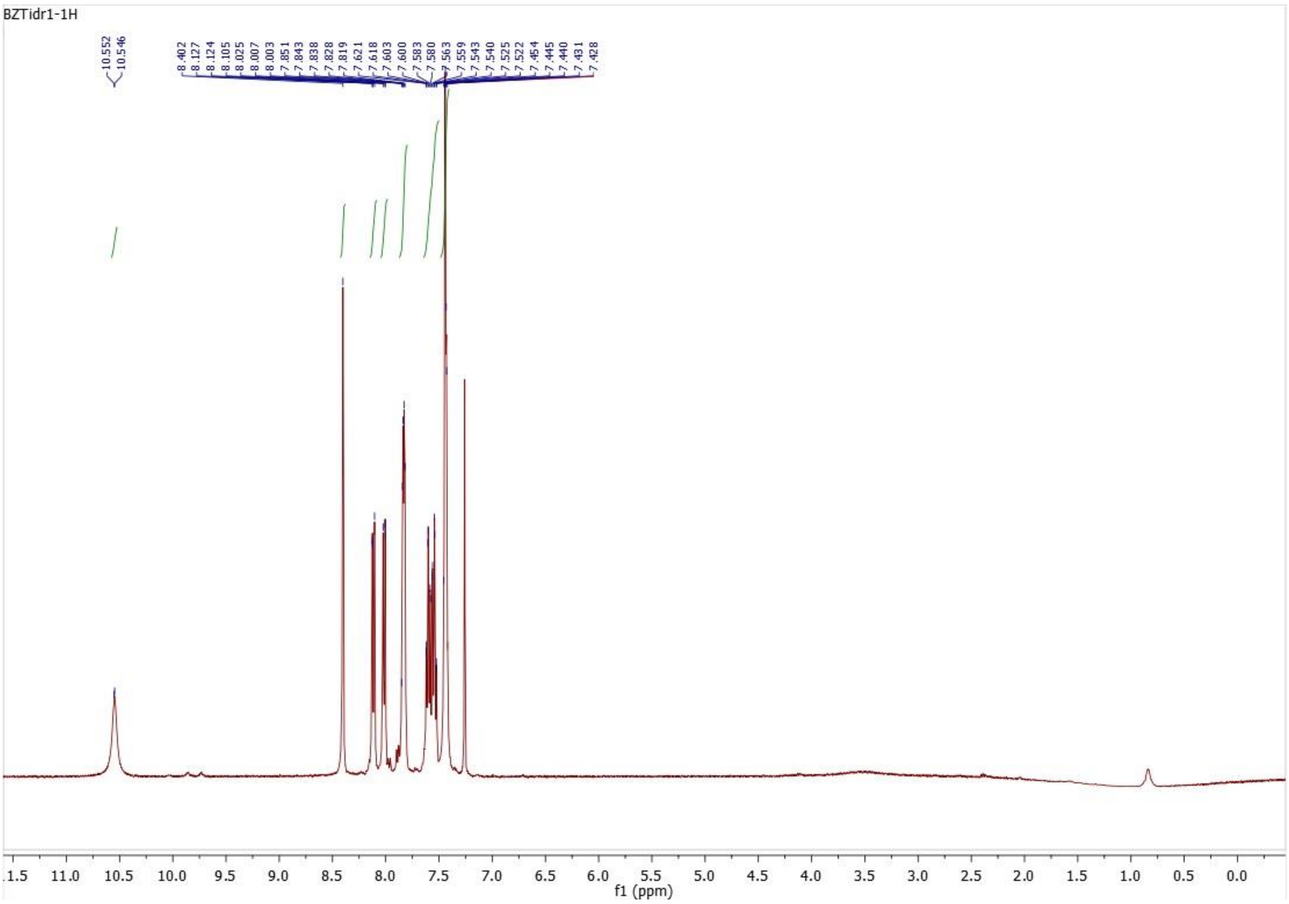

Figure S3. ^{1}H-NMR spectrum of compound **BZTidr1**.

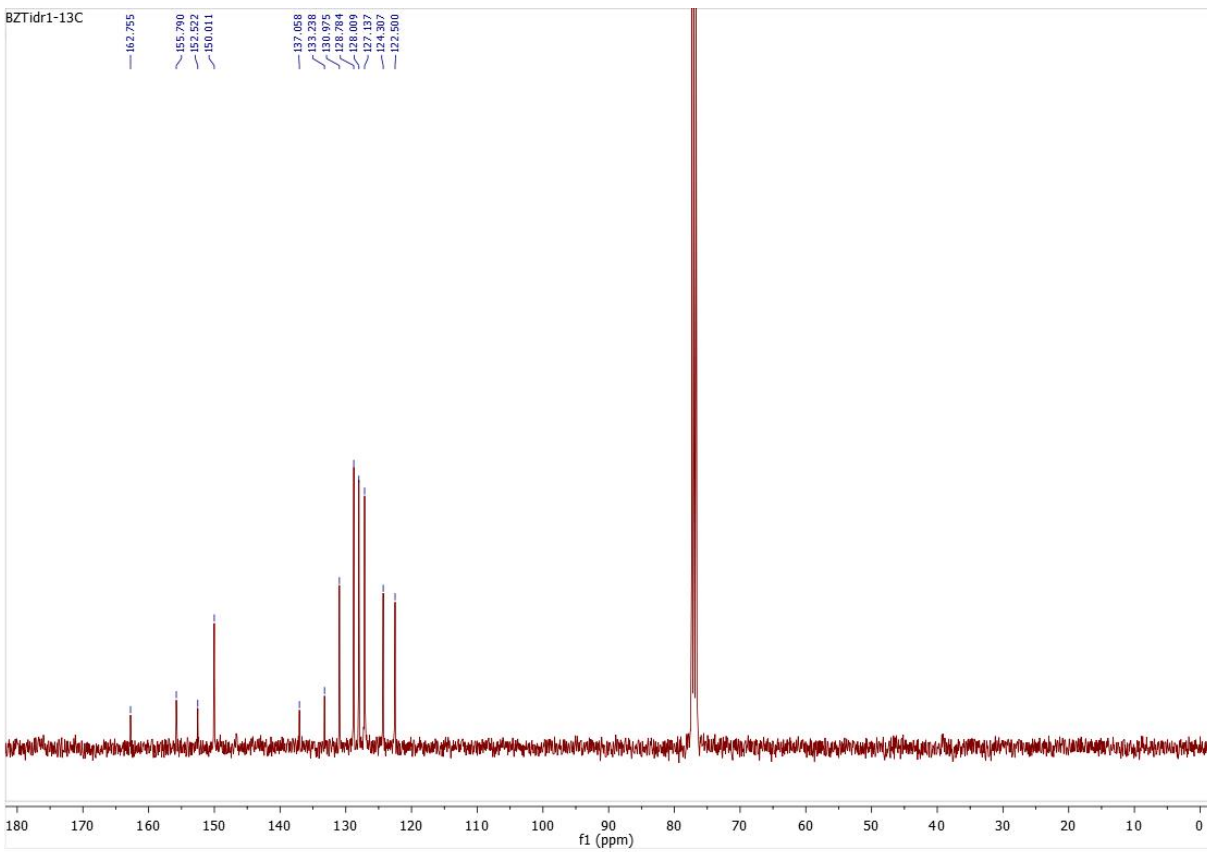

Figure S4. ^{13}C-NMR spectrum of compound **BZTidr1**.

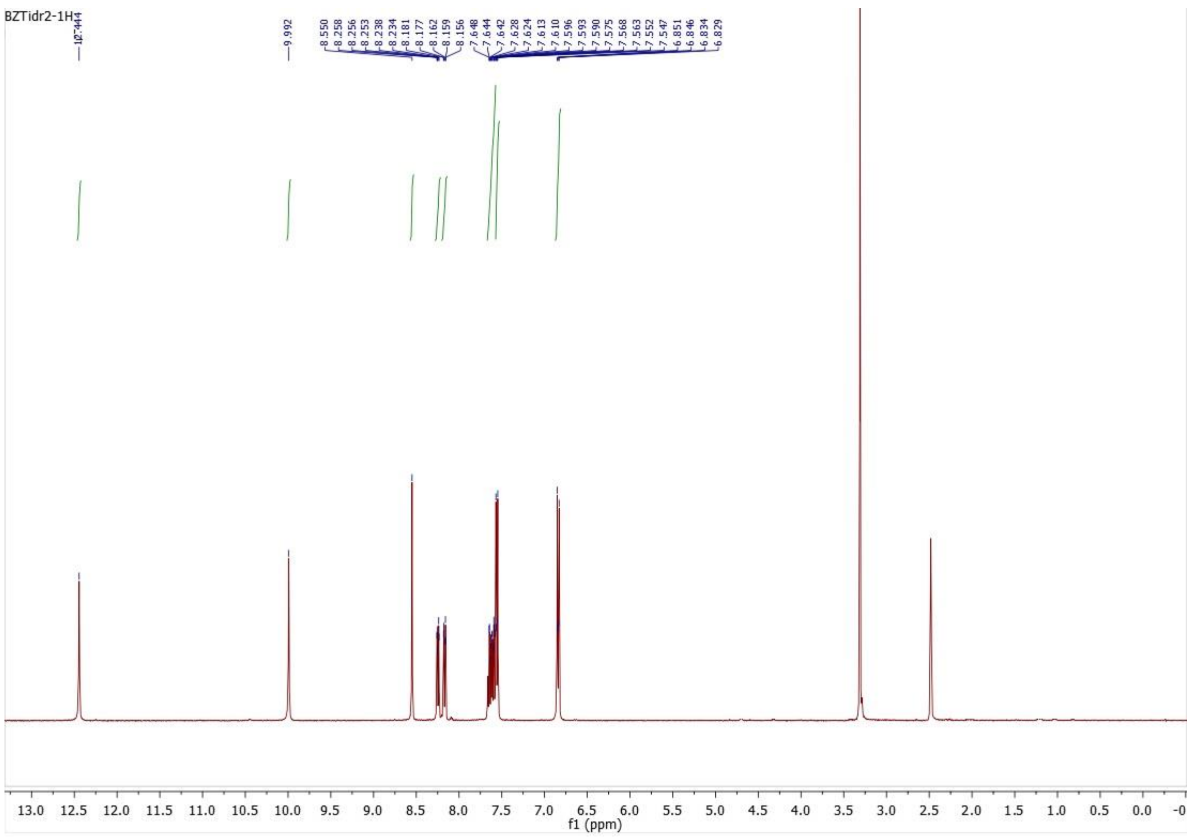

Figure S5. ^{1}H-NMR spectrum of compound **BZTidr2**.

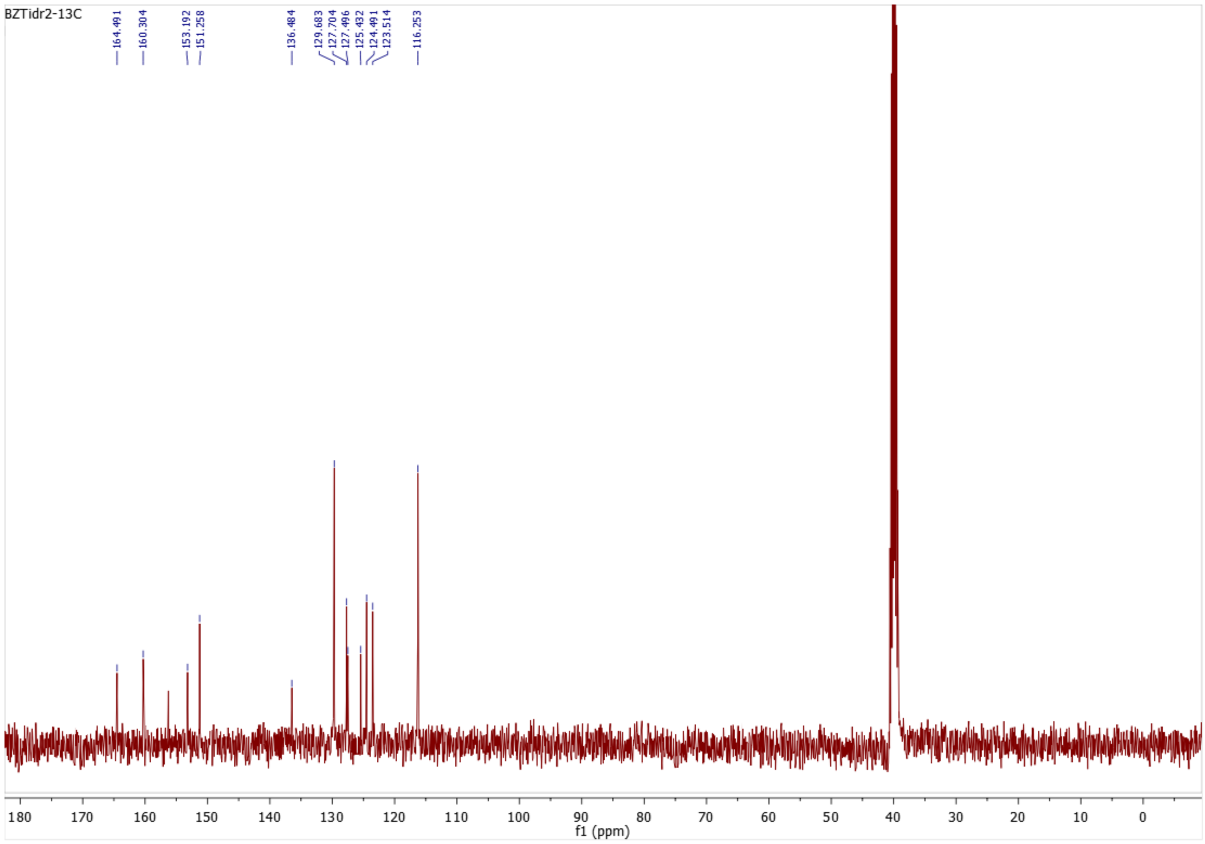

Figure S6. ^{13}C-NMR spectrum of compound **BZTidr2**.

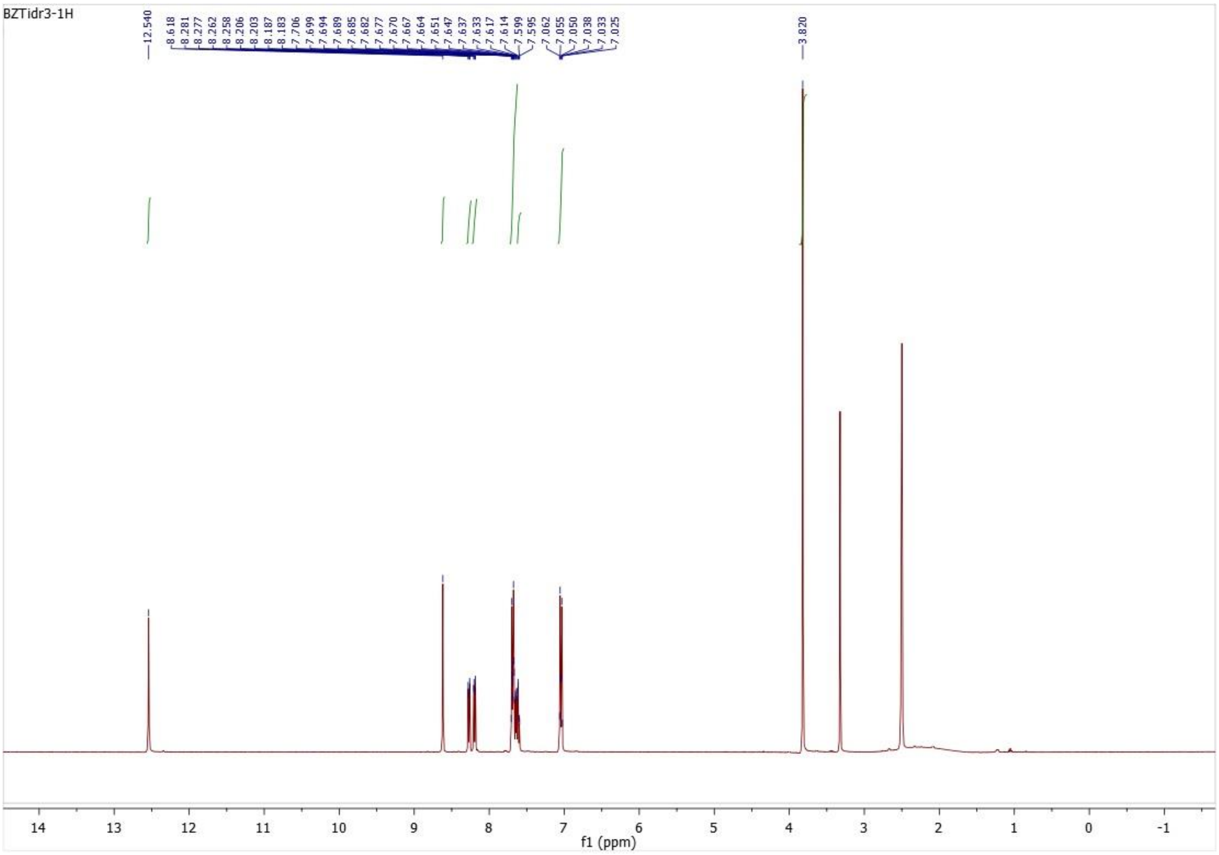

Figure S7. ^{1}H-NMR spectrum of compound **BZTidr3**.

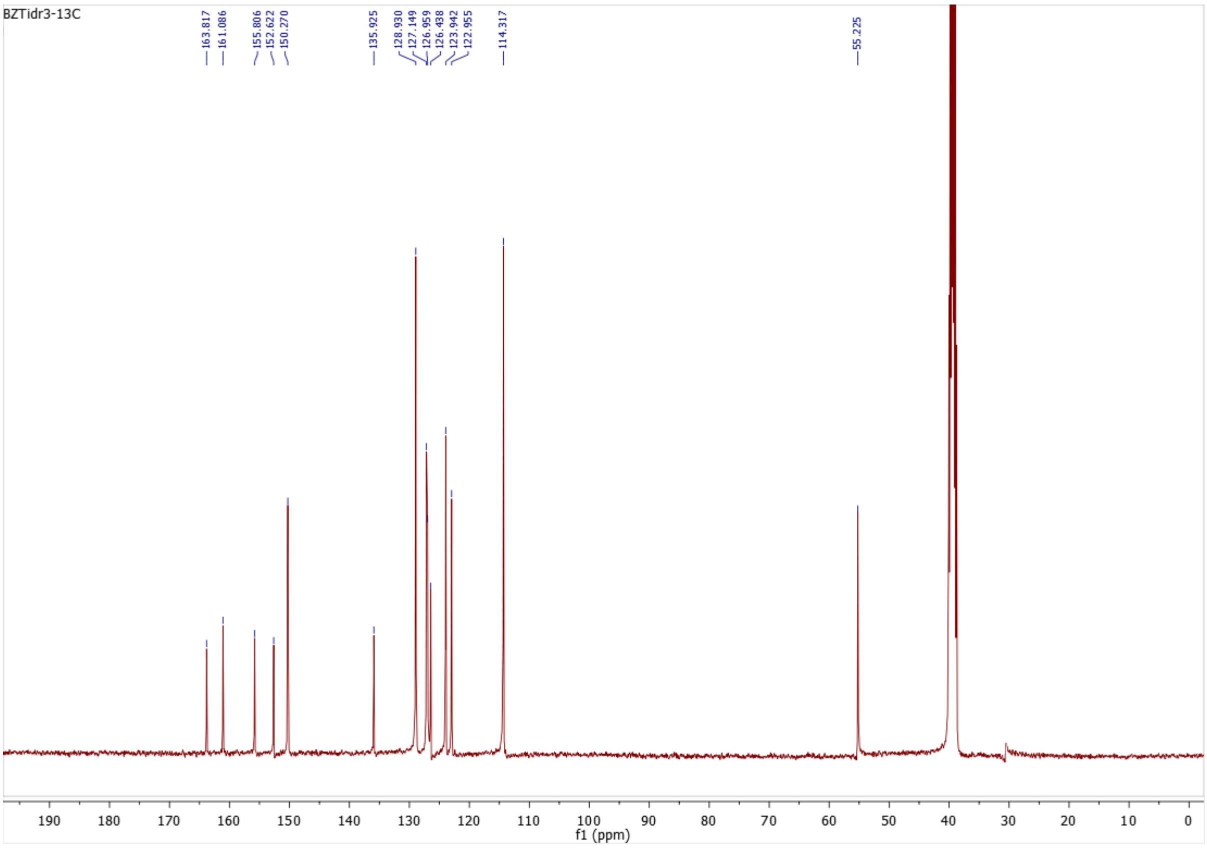

Figure S8. ^{13}C-NMR spectrum of compound **BZTidr3**.

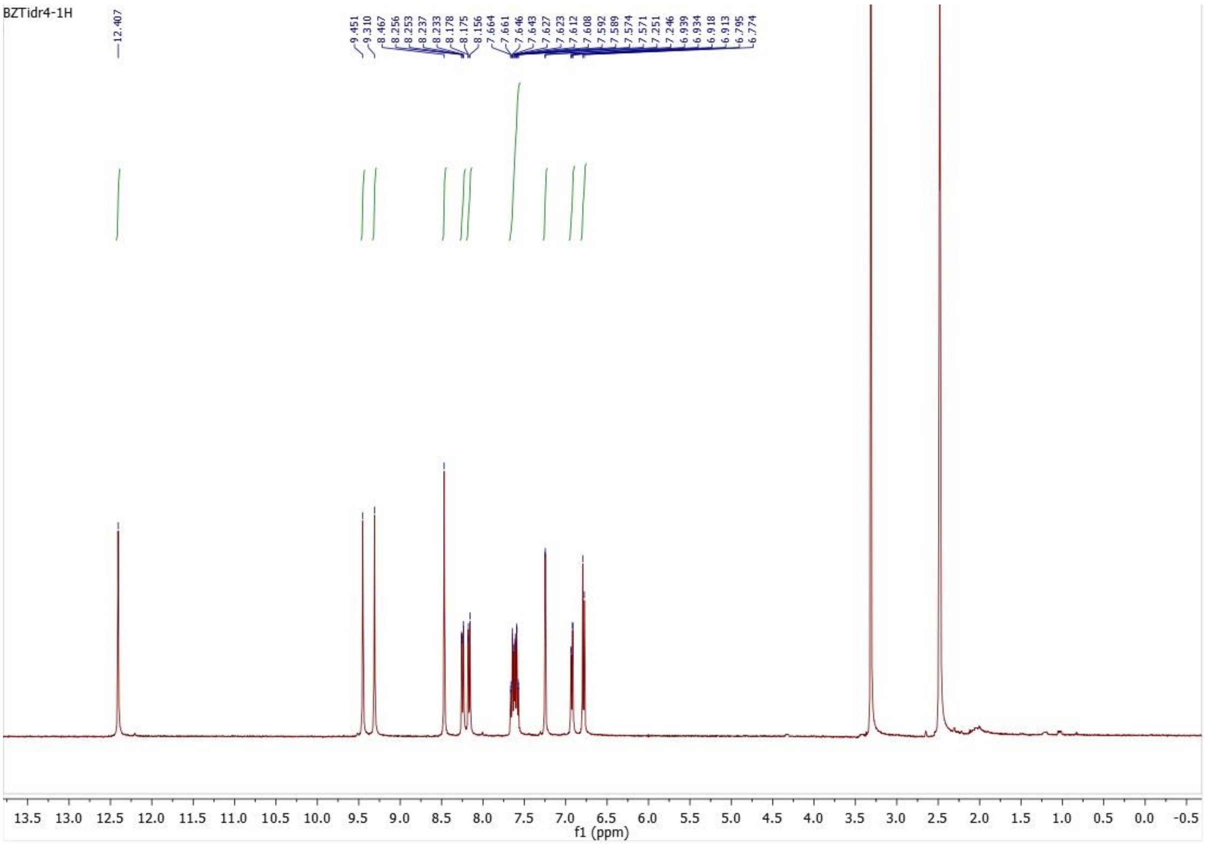

Figure S9. ^{1}H-NMR spectrum of compound **BZTidr4**.

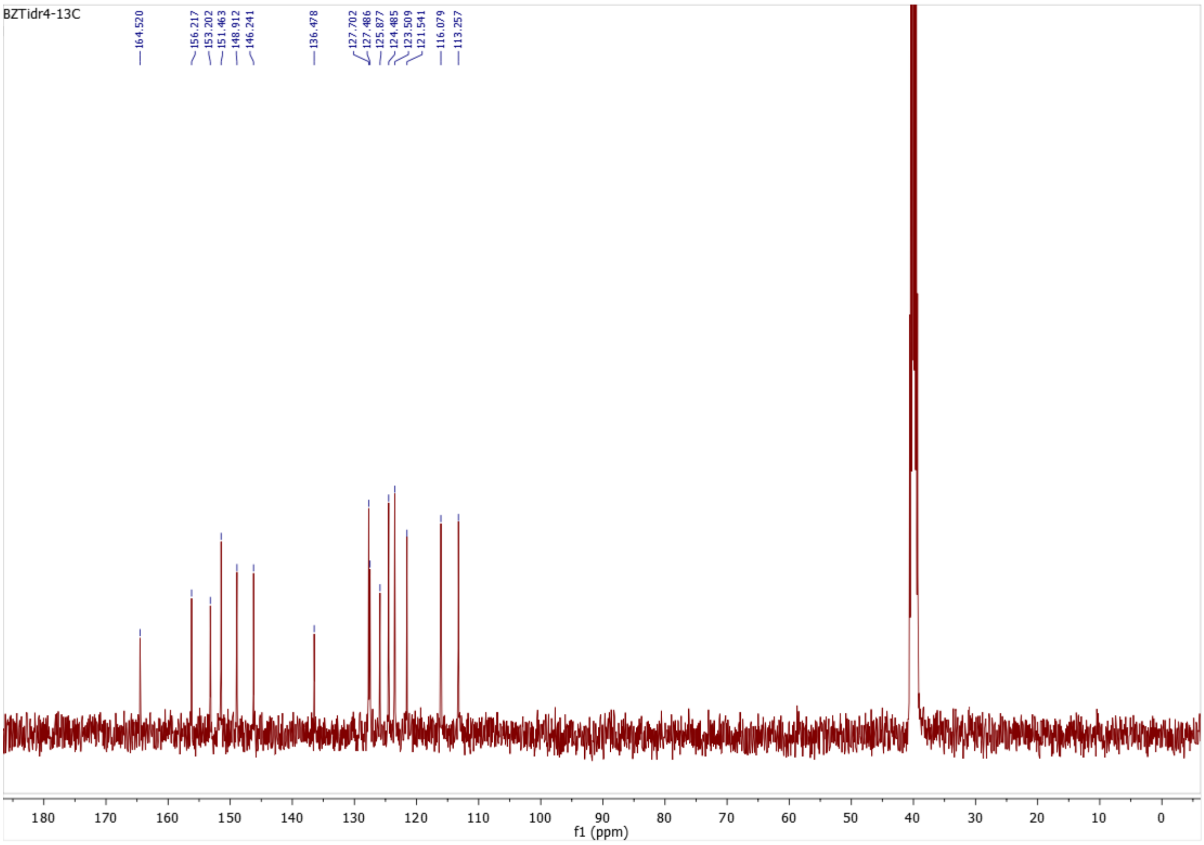

Figure S10. ^{13}C-NMR spectrum of compound **BZTidr4**.

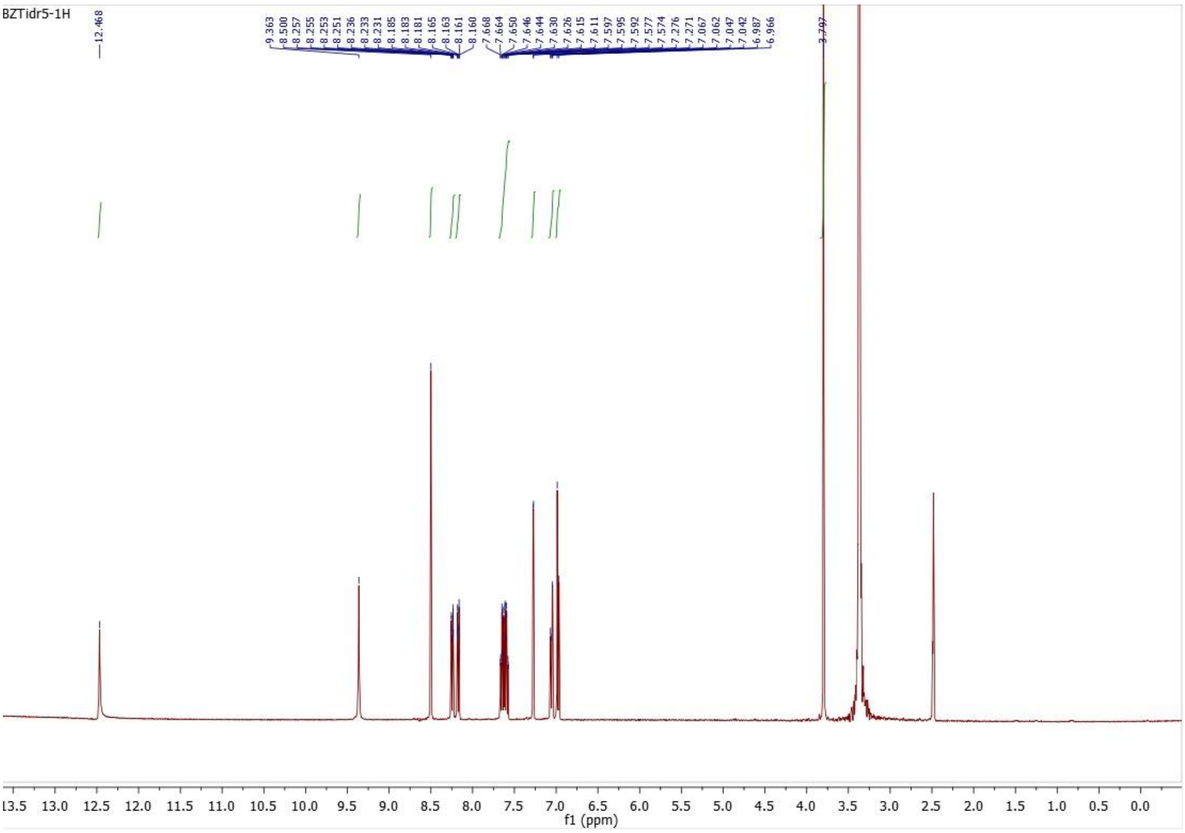

Figure S11. ^{1}H-NMR spectrum of compound **BZTidr5**.

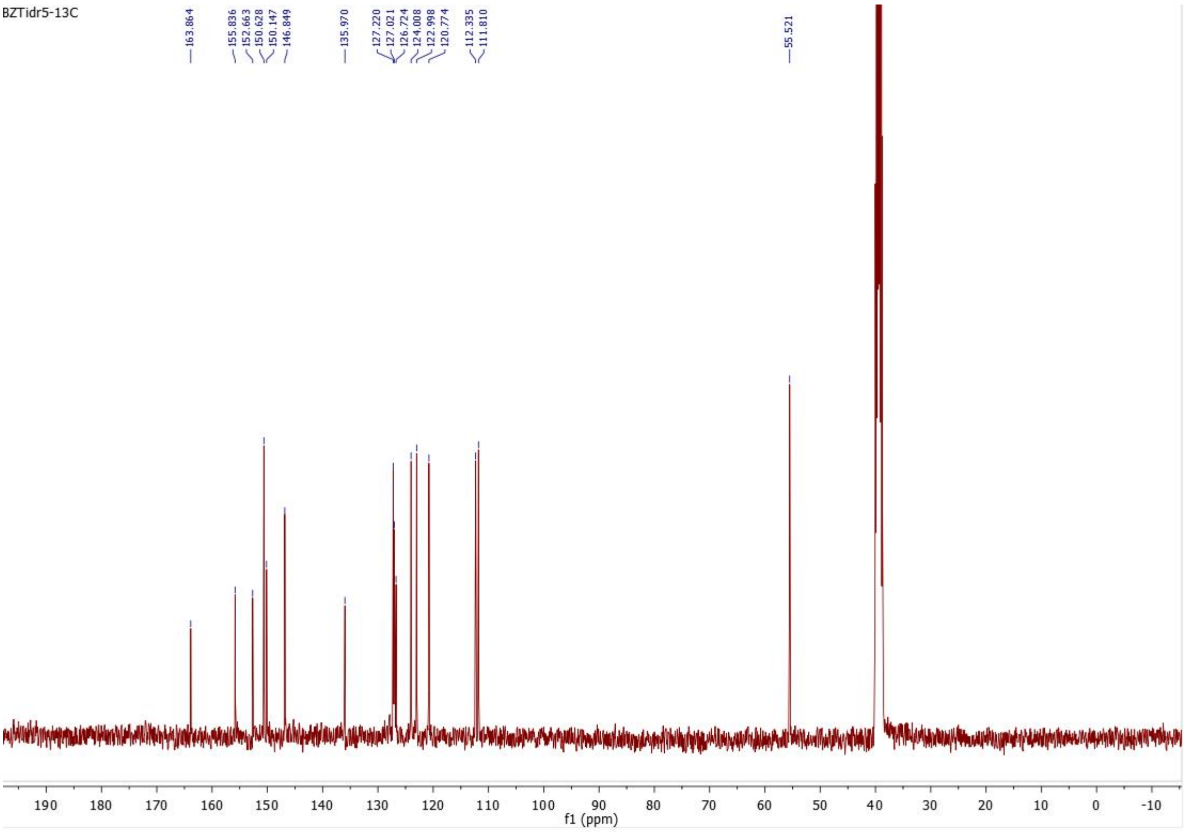

Figure S12. ^{13}C-NMR spectrum of compound **BZTidr5**.

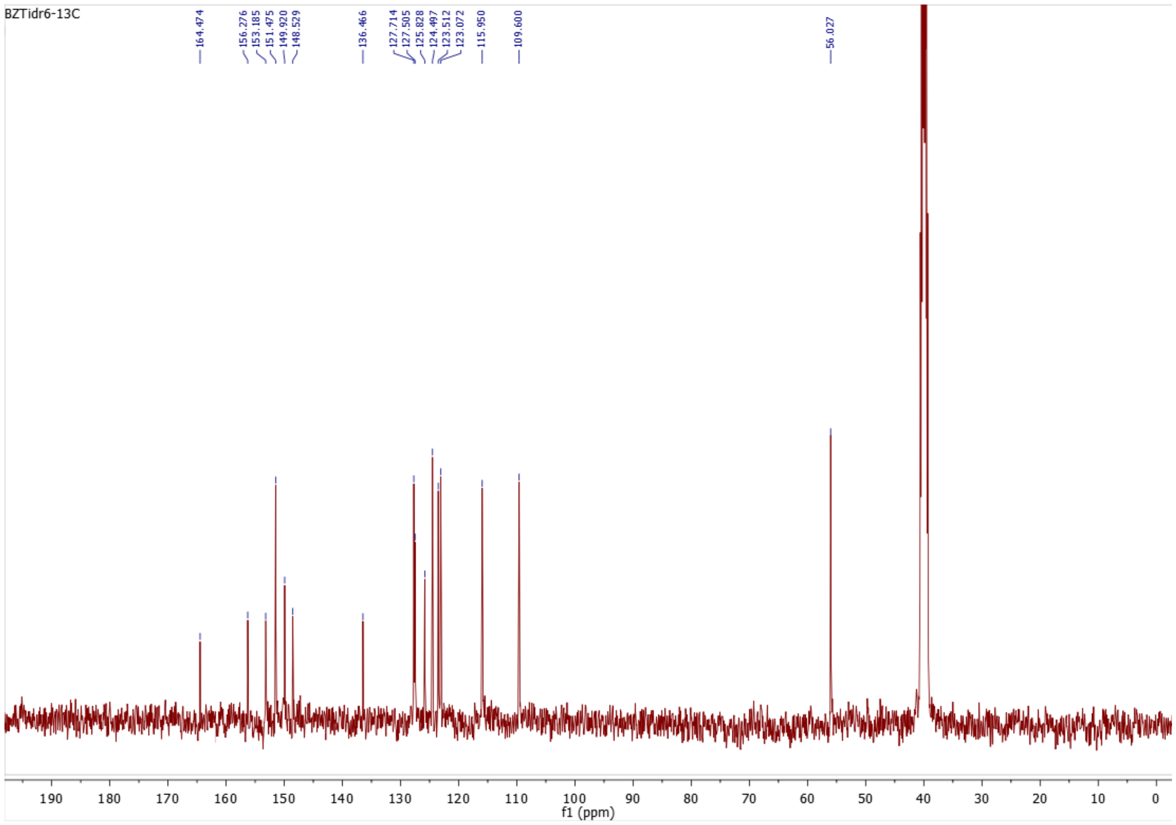

Figure S13. ^{1}H-NMR spectrum of compound **BZTidr6**.

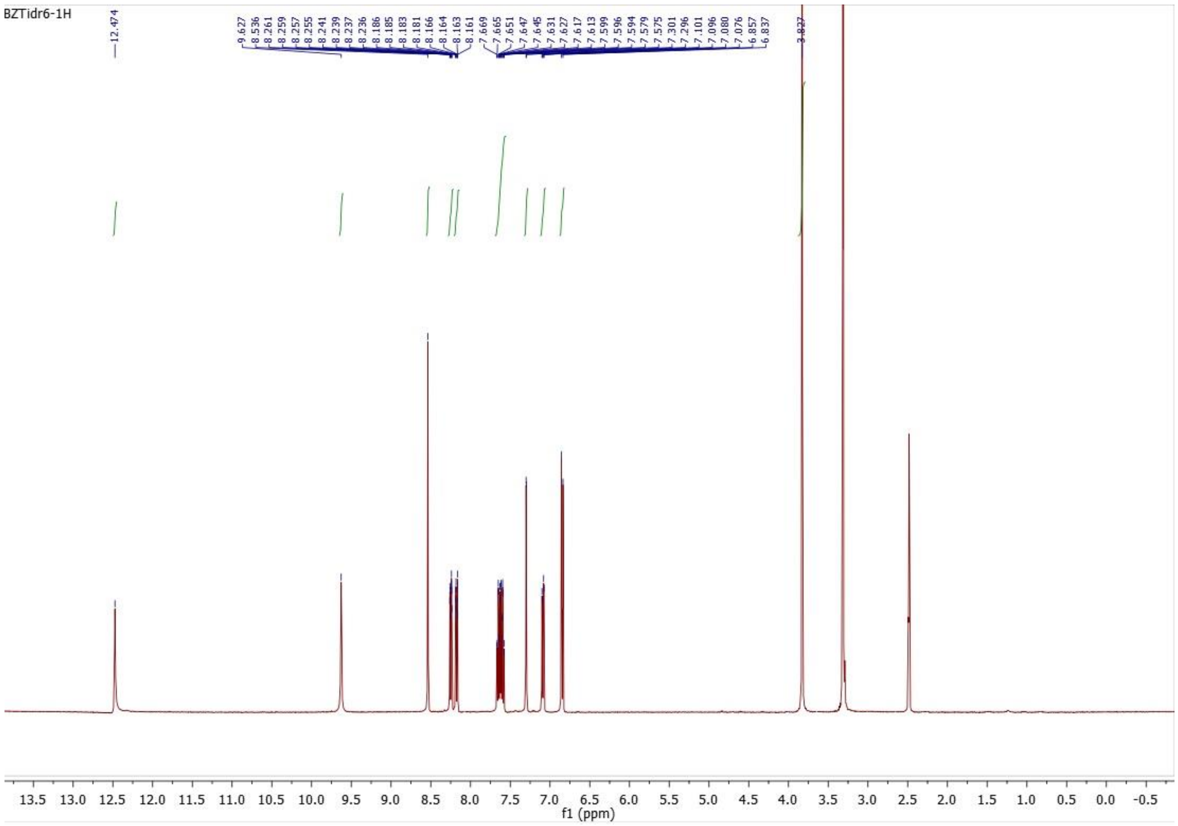

Figure S14. ^{13}C-NMR spectrum of compound **BZTidr6**.

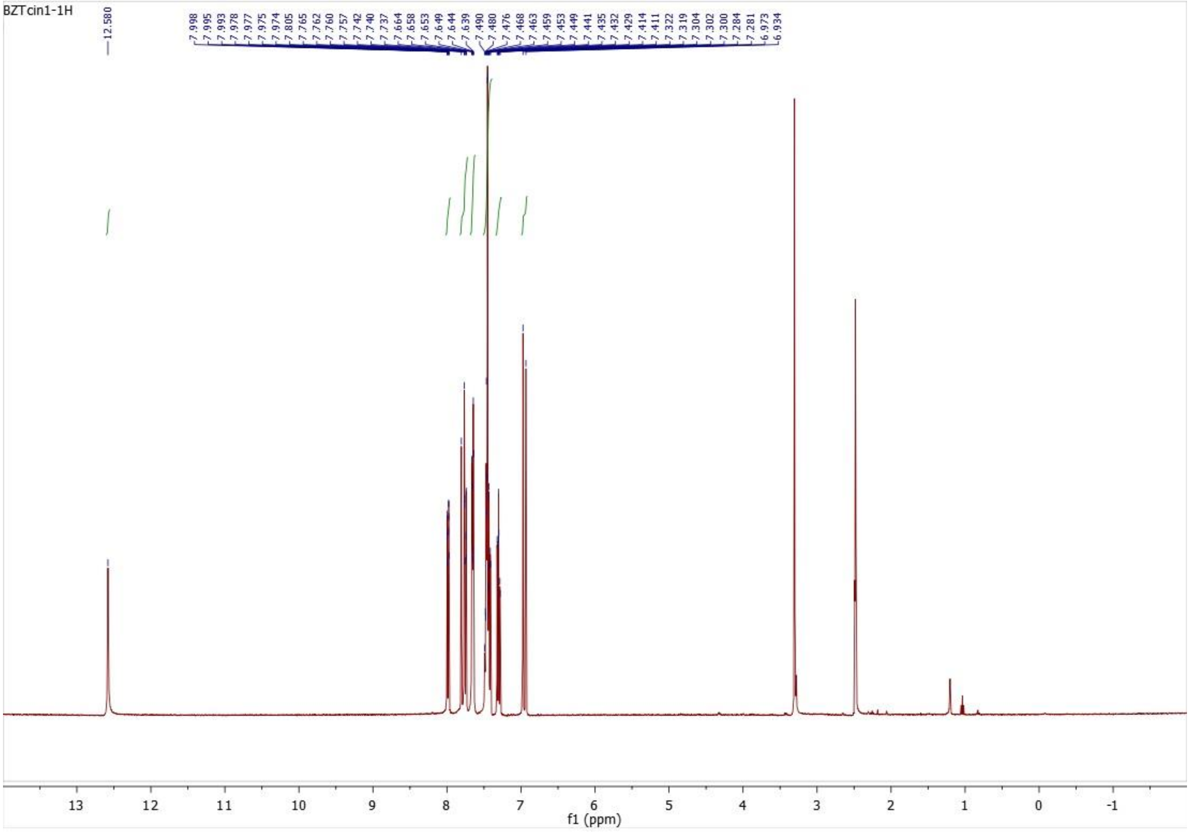

Figure S15. ^{1}H-NMR spectrum of compound **BZTcin1**.

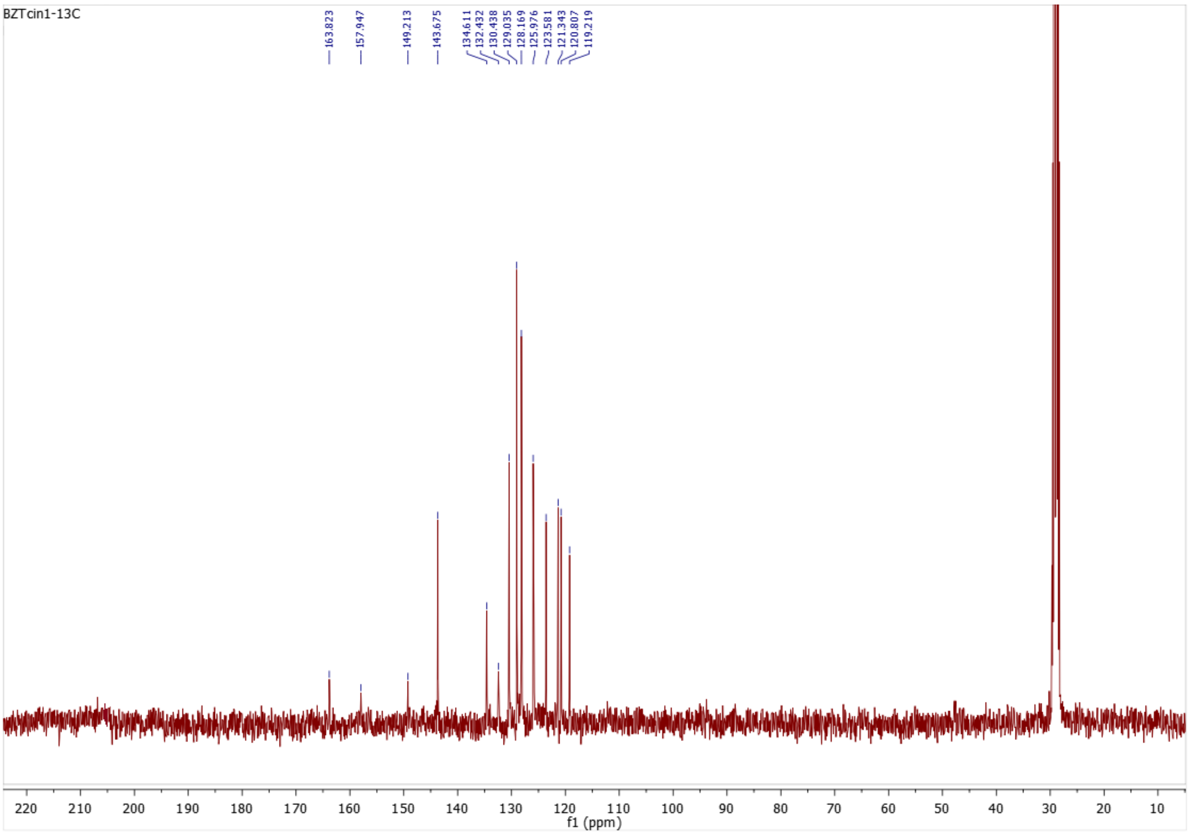

Figure S16. ^{13}C-NMR spectrum of compound **BZTcin1**.

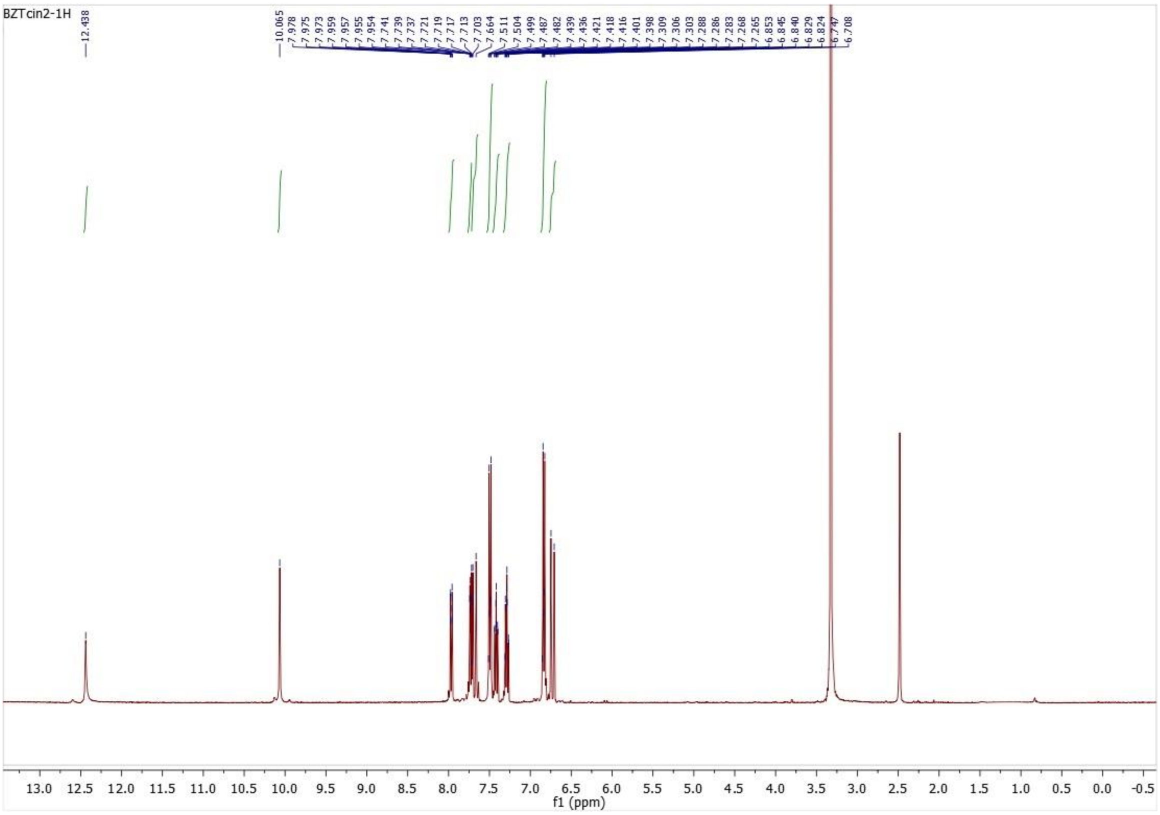

Figure S17. ^{1}H-NMR spectrum of compound **BZTcin2**.

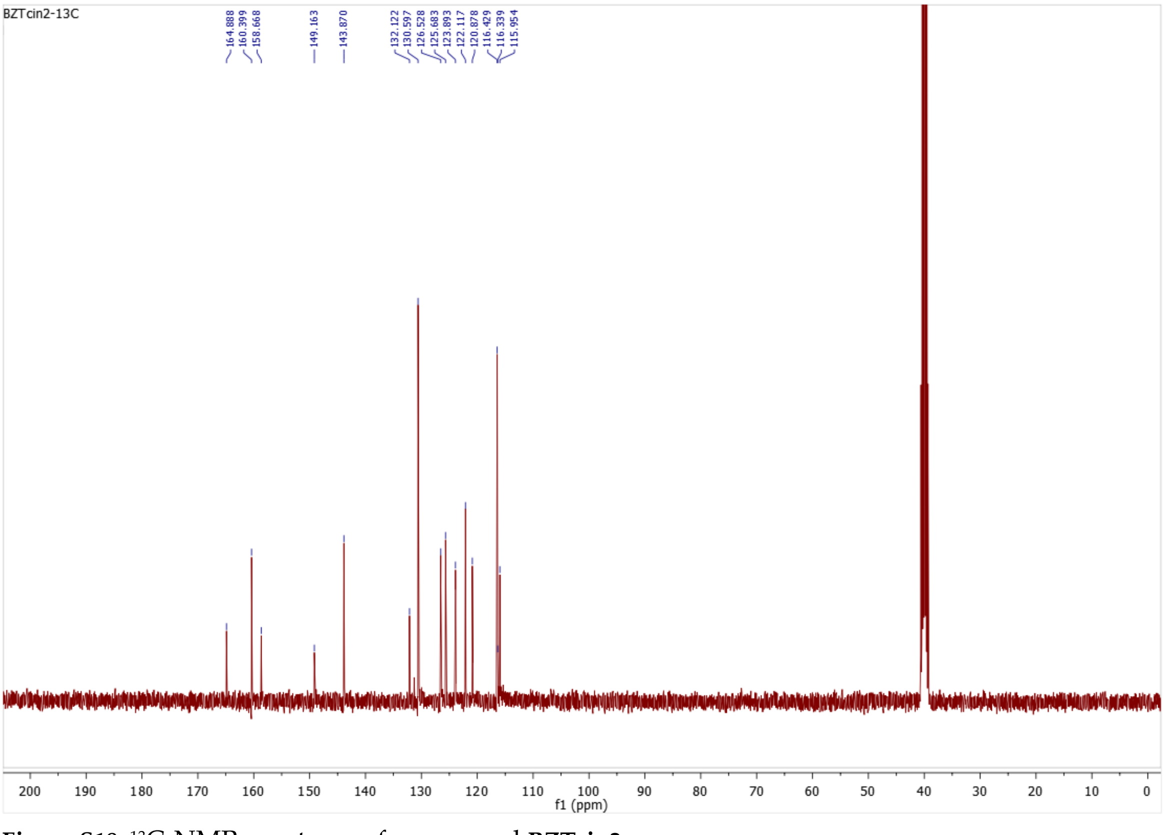

Figure S18. ^{13}C-NMR spectrum of compound **BZTcin2**.

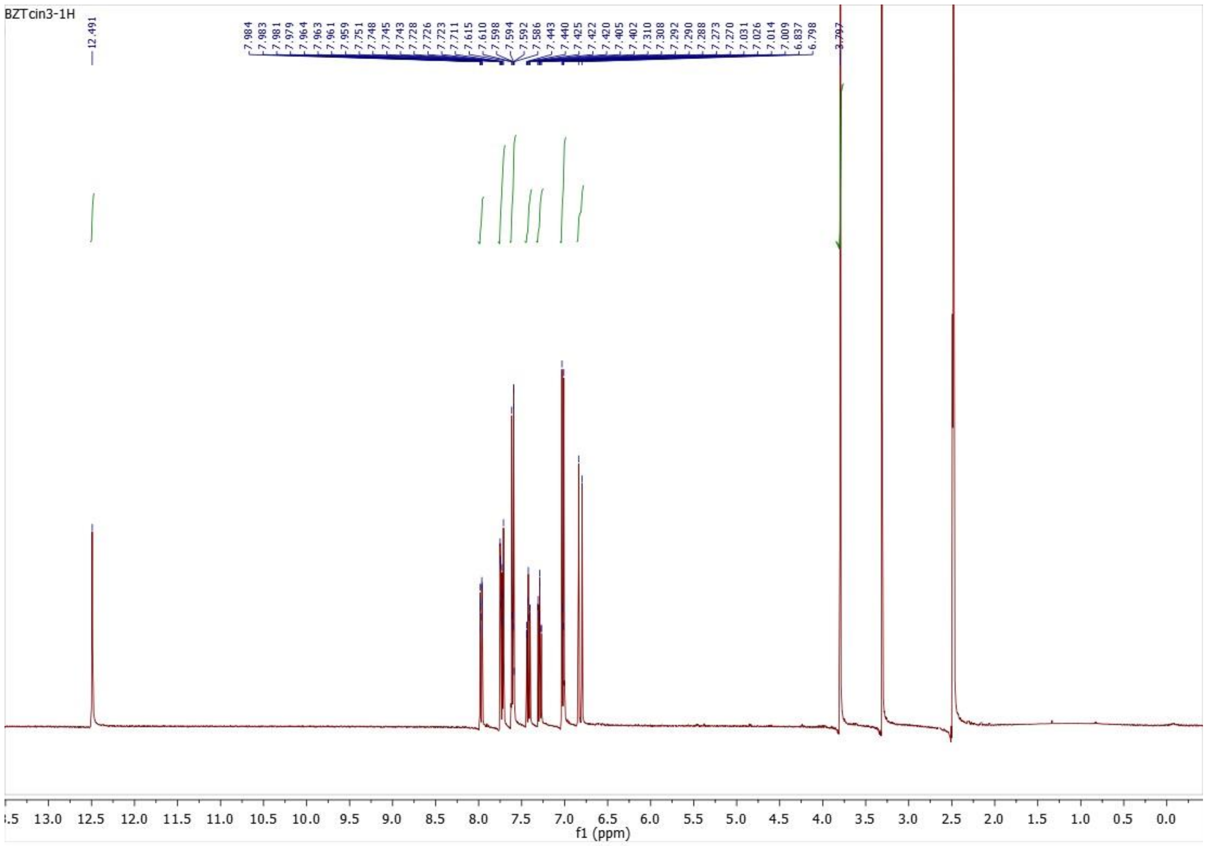

Figure S19. ^{1}H-NMR spectrum of compound **BZTcin3**.

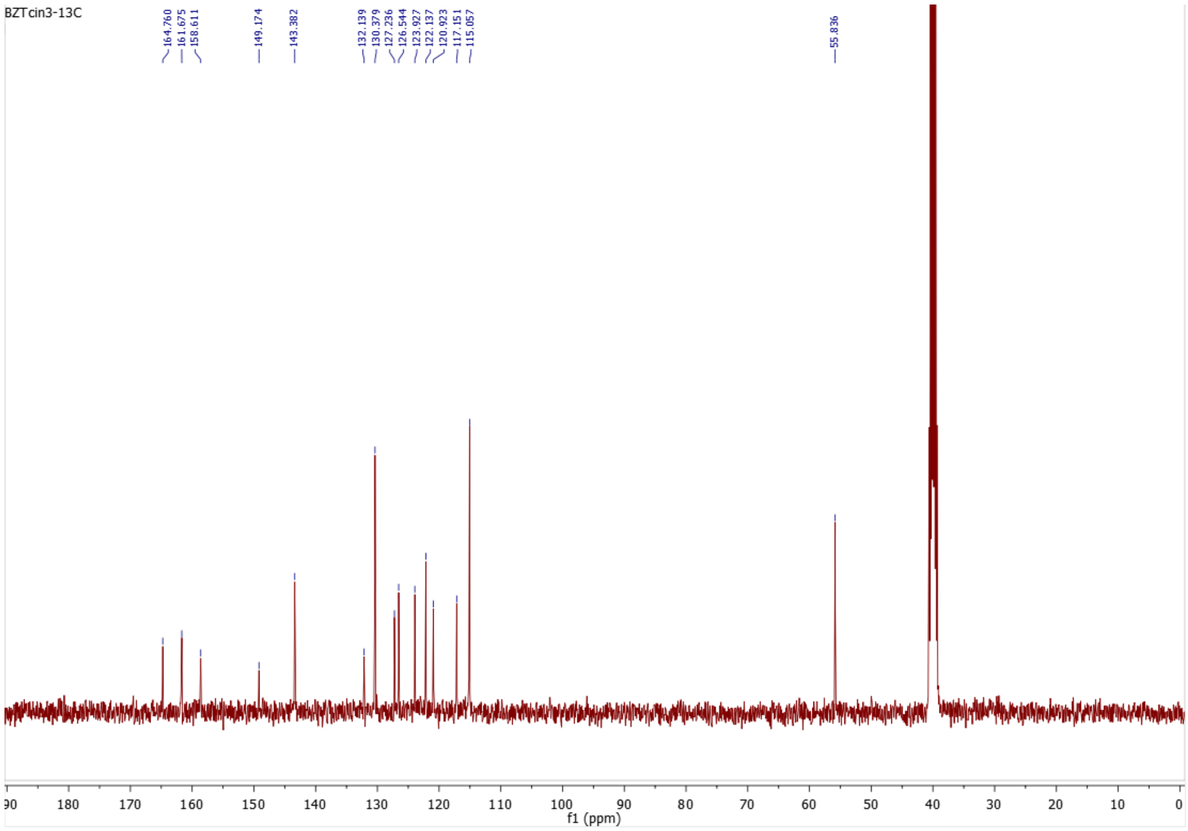

Figure S20. ^{13}C-NMR spectrum of compound **BZTcin3**.

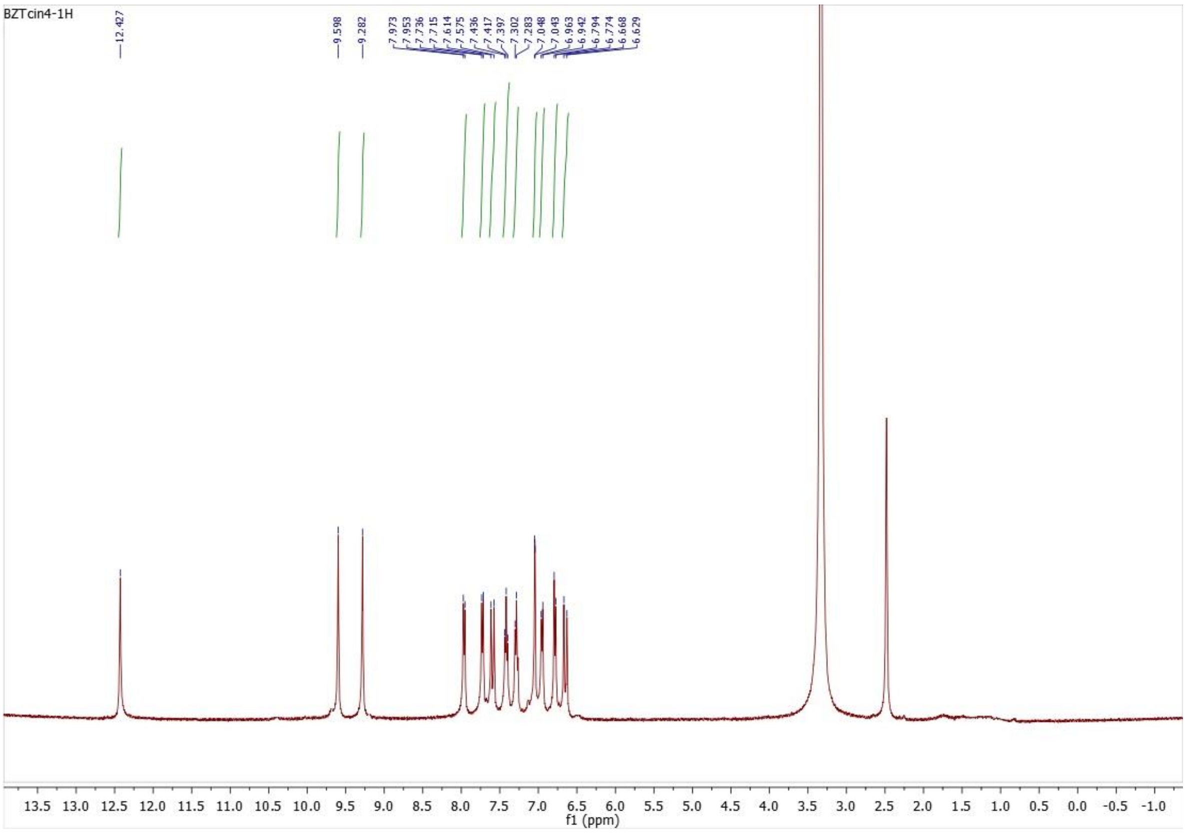

Figure S21. ^{1}H-NMR spectrum of compound **BZTcin4**.

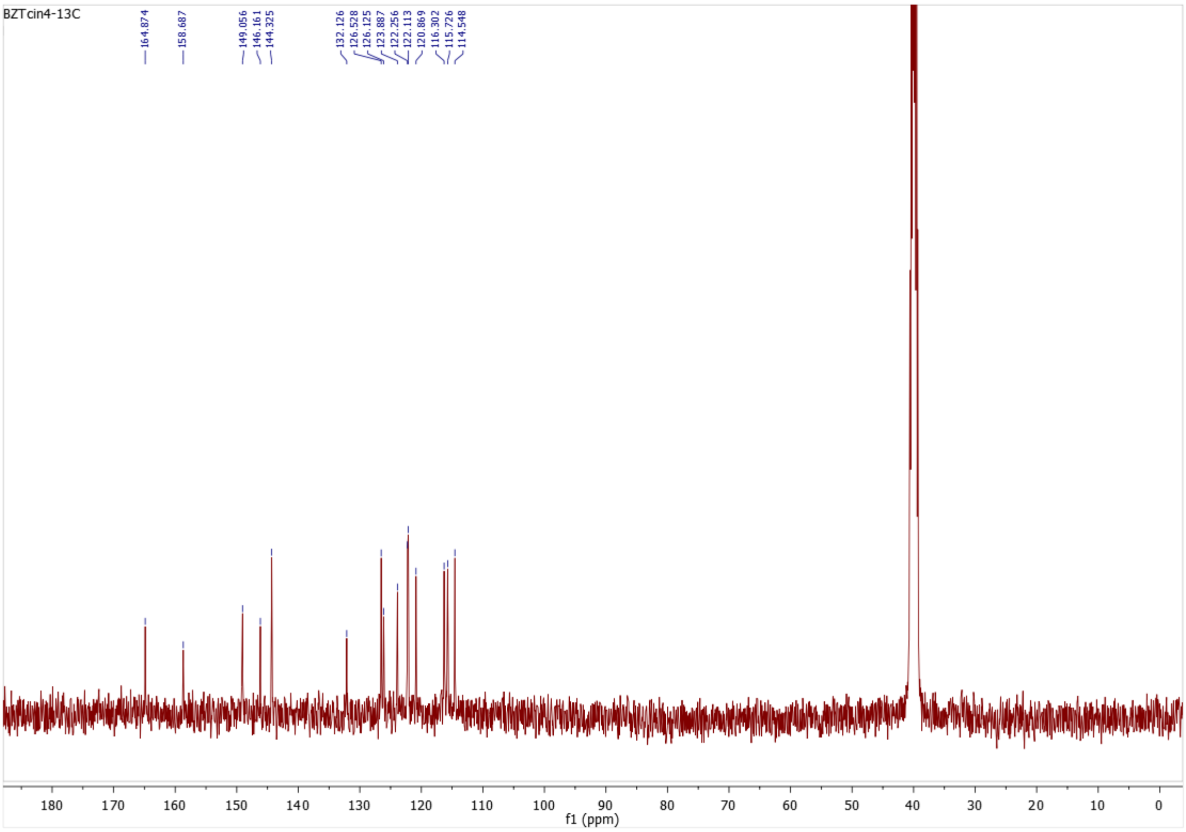

Figure S22. ^{13}C-NMR spectrum of compound **BZTcin4**.

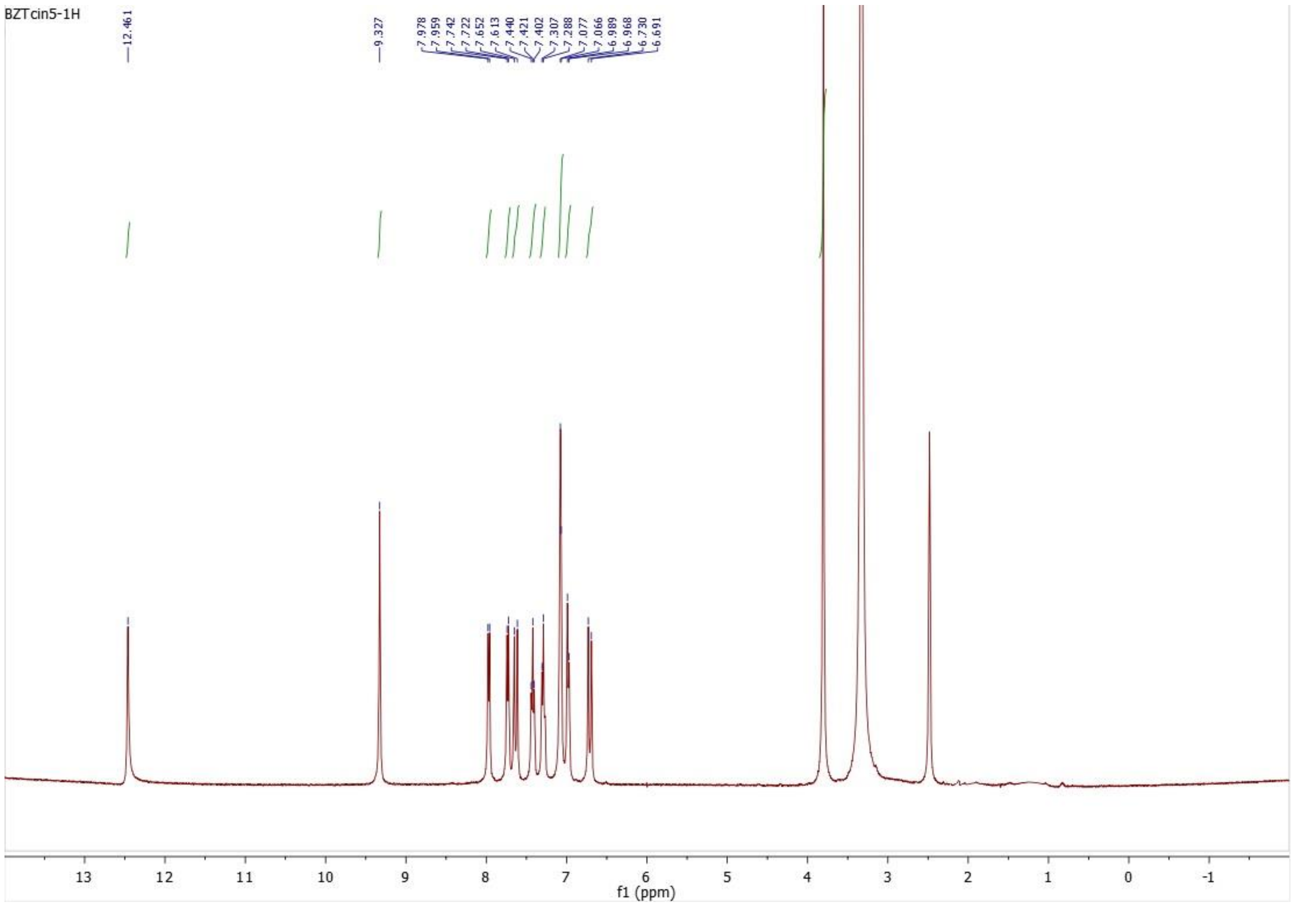

Figure S23. ^{1}H-NMR spectrum of compound **BZTcin5**.

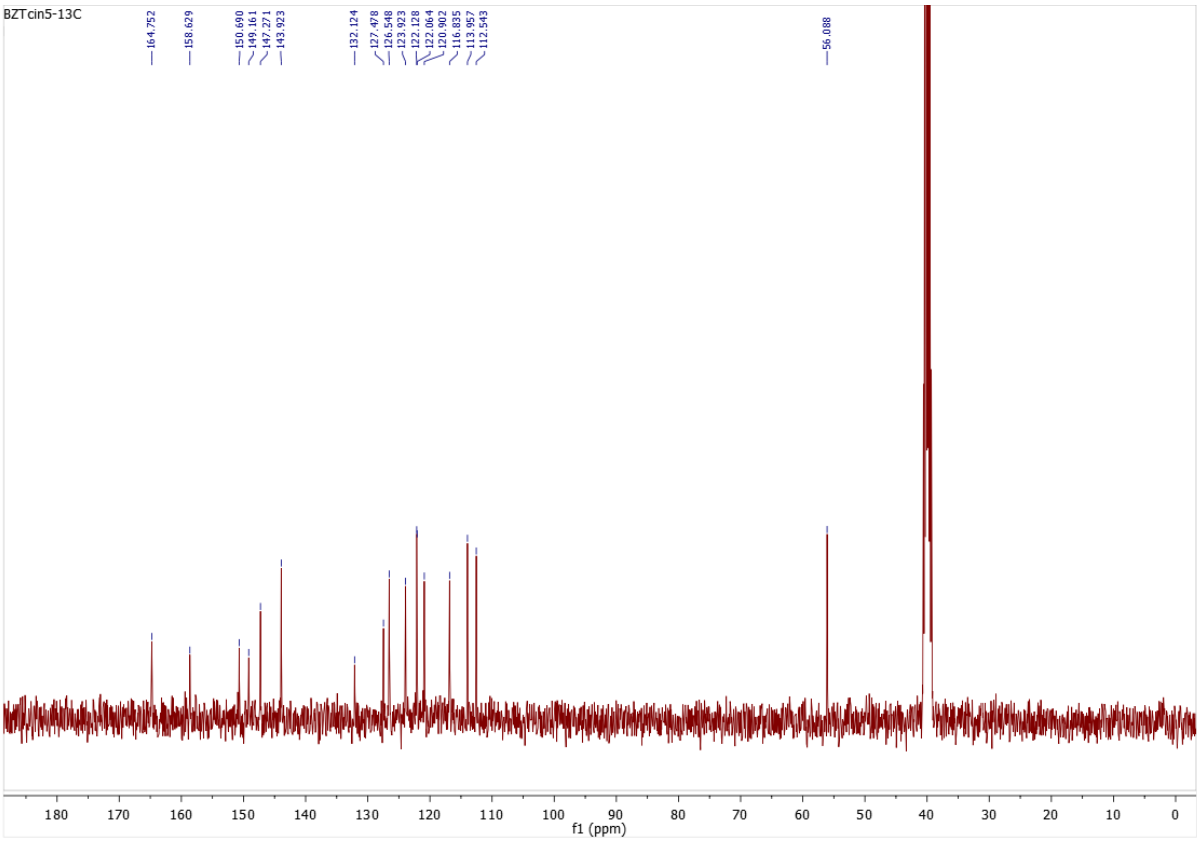

Figure S24. ^{13}C-NMR spectrum of compound **BZTcin5**.

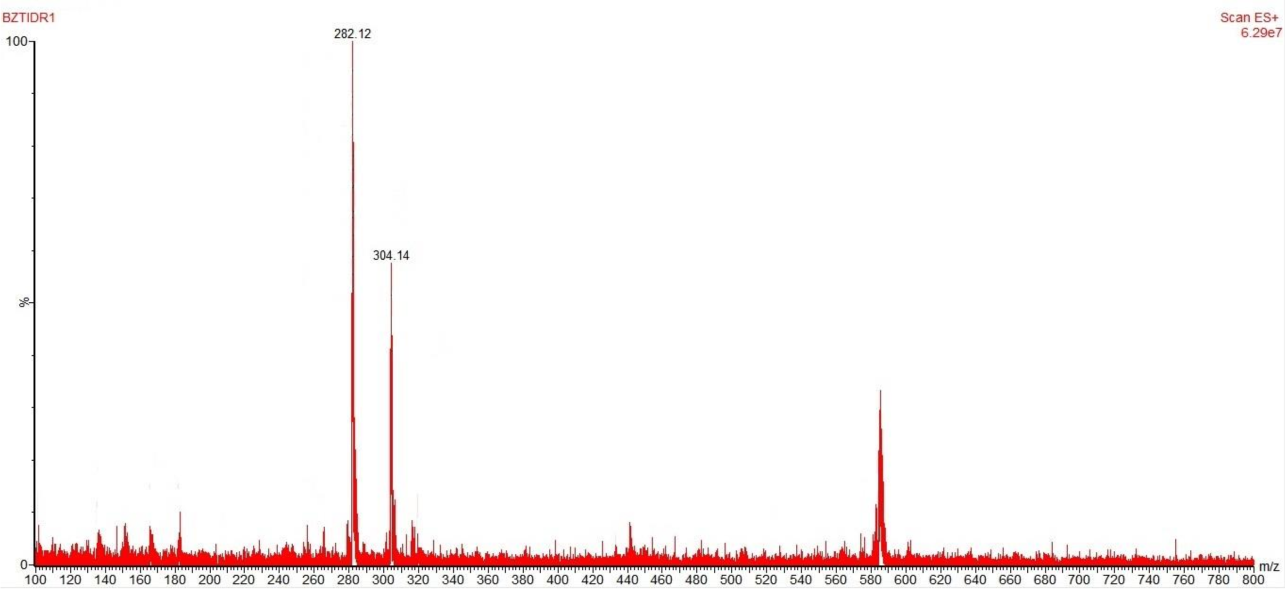

Figure S25. Mass spectrum of (E)-N'-benzylidenebenzo[d]thiazole-2-carbohydrazide (**BZTidr1)**.

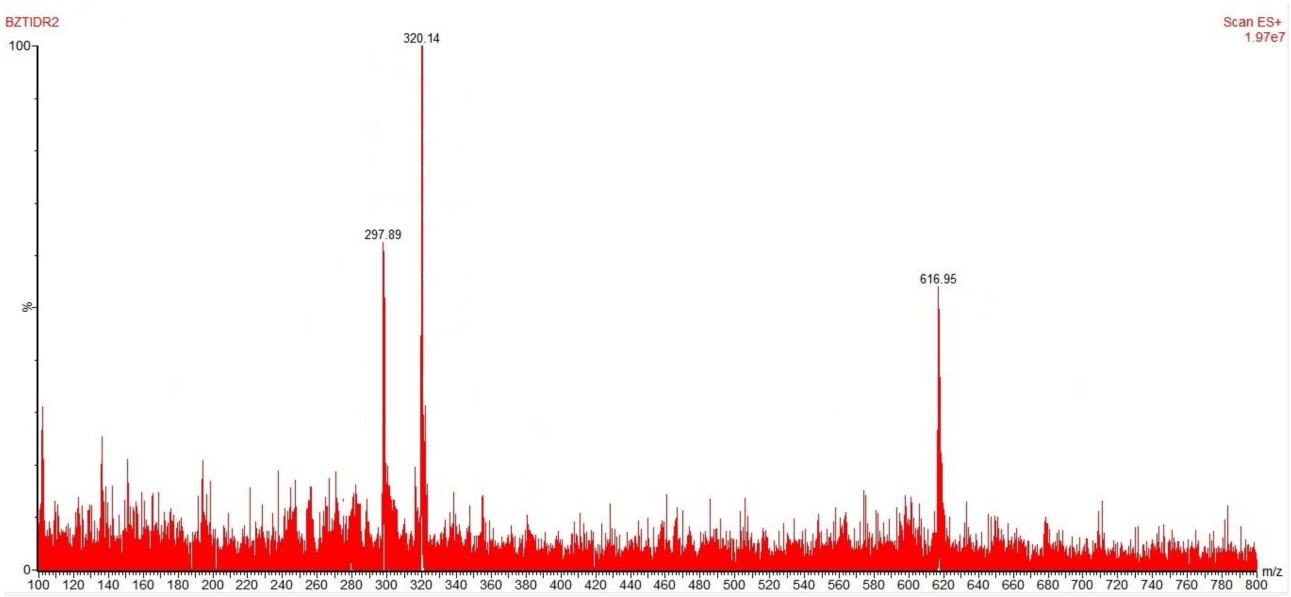

Figure S26. Mass spectrum of (E)-N'-(4-hydroxybenzylidene)benzo[d]thiazole-2-carbohydrazide (**BZTidr2**).

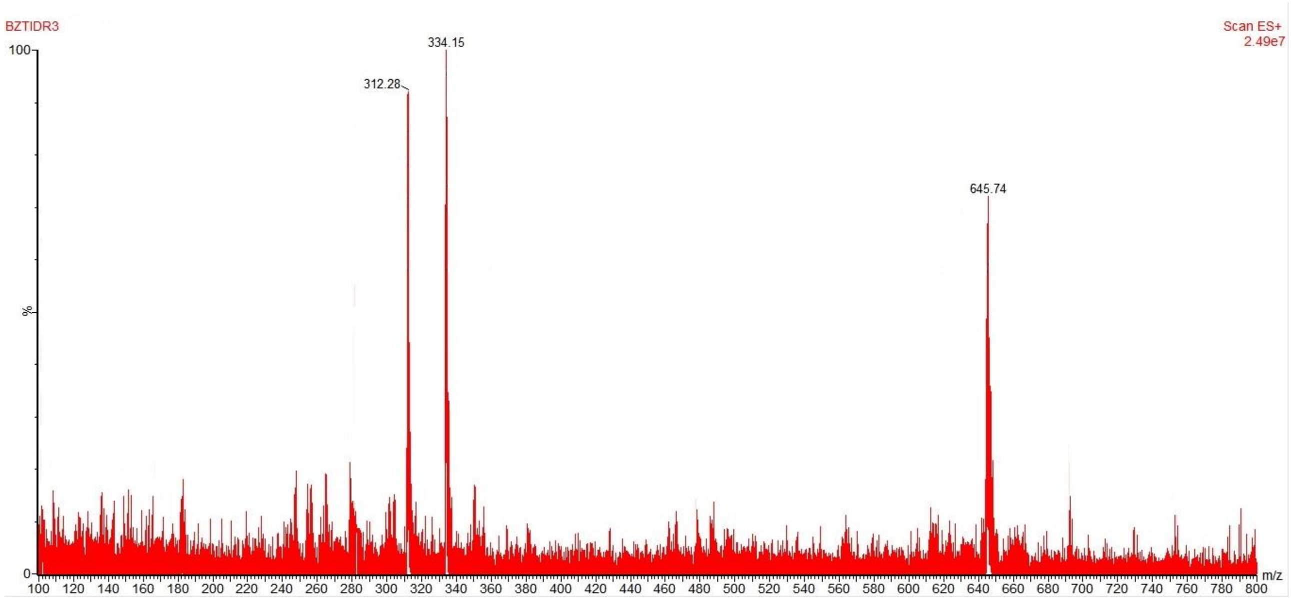

Figure S27. Mass spectrum of (E)-N'-(4-methoxybenzylidene)benzo[d]thiazole-2-carbohydrazide (**BZTidr3**).

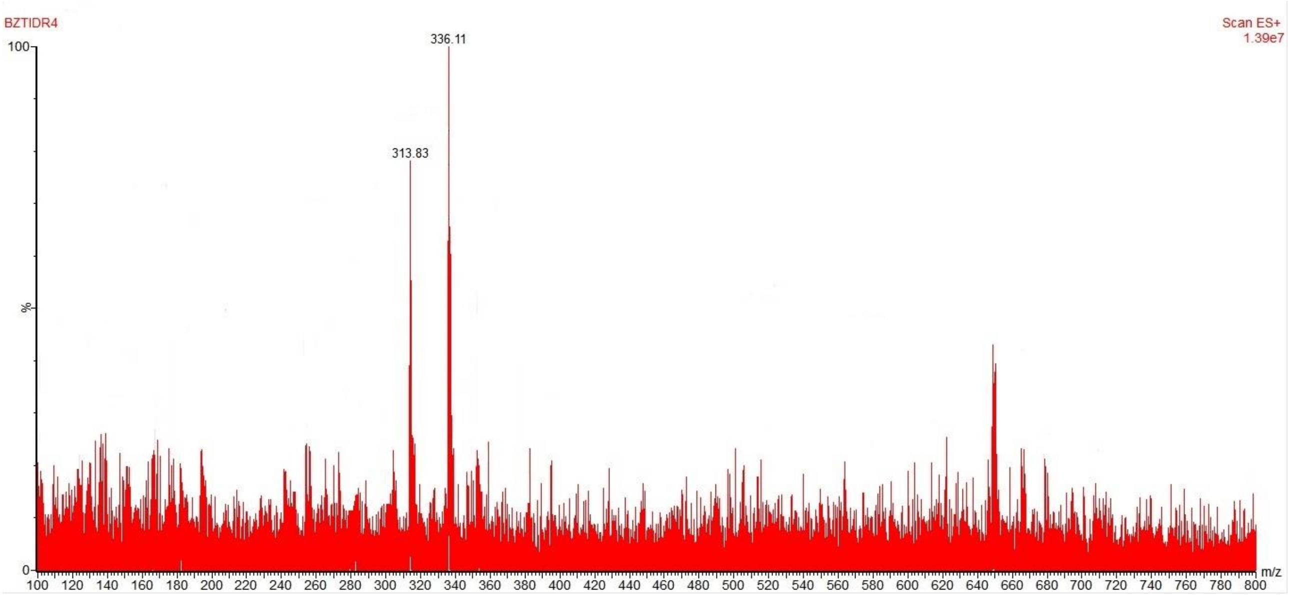

Figure S28. Mass spectrum of (E)-N'-(3,4-dihydroxybenzylidene)benzo[d]thiazole-2-carbohydrazide (**BZTidr4**).

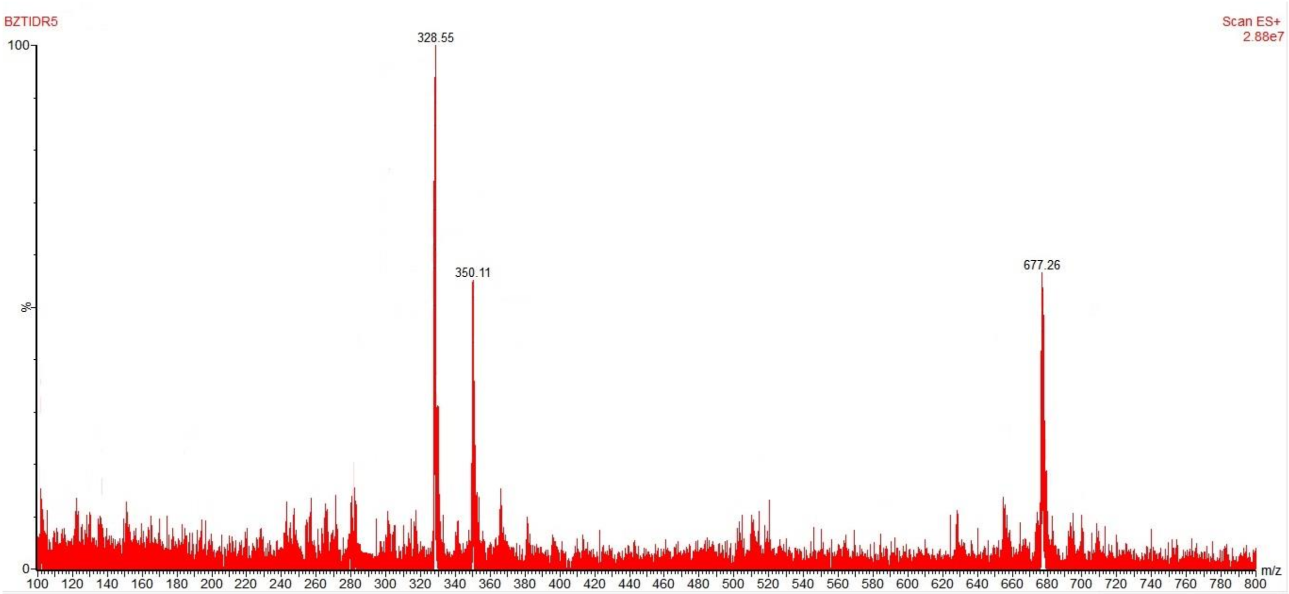

Figure S29. Mass spectrum of (E)-N'-(3-hydroxy-4-methoxybenzylidene)benzo[d]thiazole-2-carbohydrazide (**BZTidr5**).

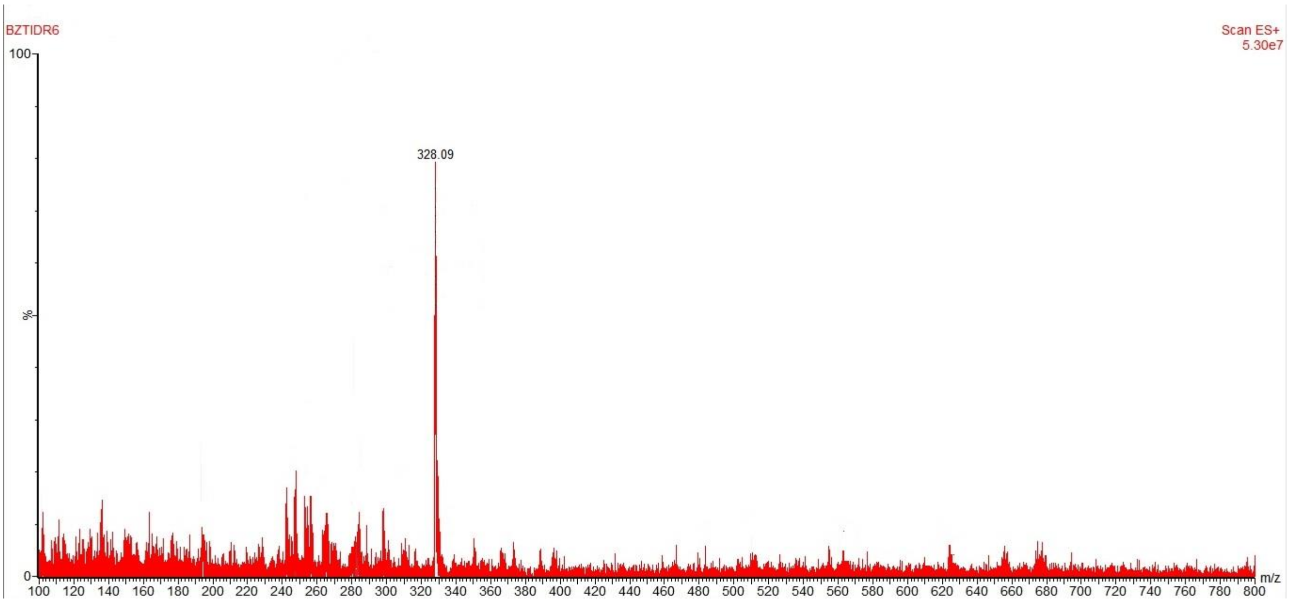

Figure S30. Mass spectrum of (E)-N'-(3-methoxy-4-hydroxybenzylidene)benzo[d]thiazole-2-carbohydrazide (**BZTidr6**).

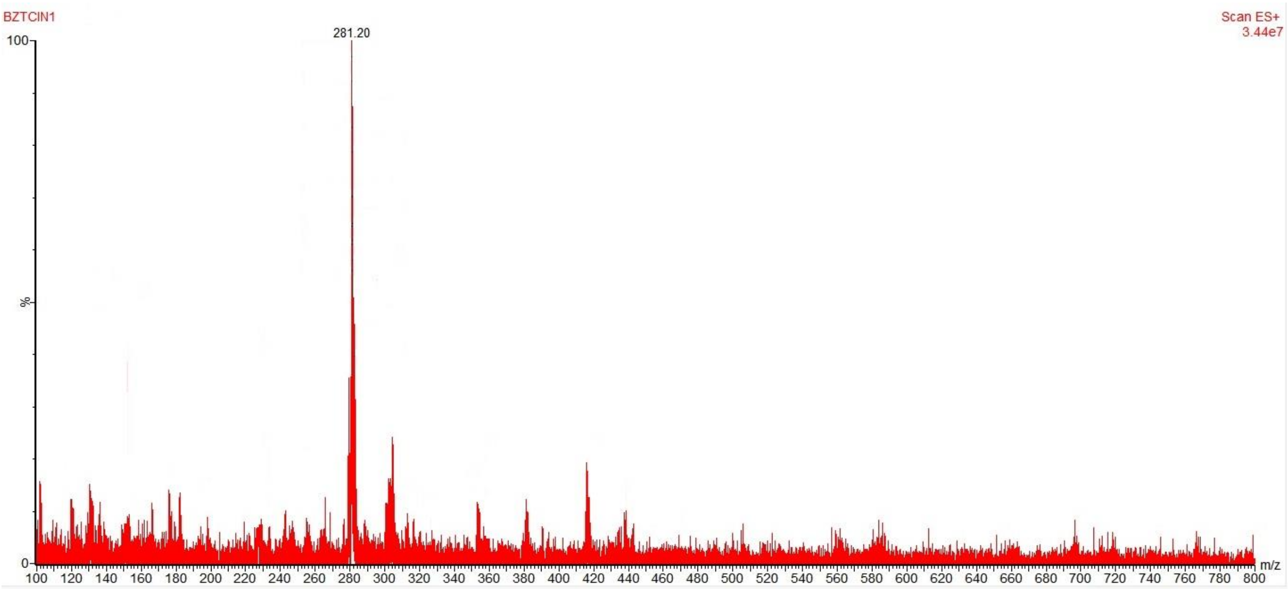

Figure S31. Mass spectrum of N-(benzo[d]thiazol-2-yl)cinnamamide (**BZTcin1**).

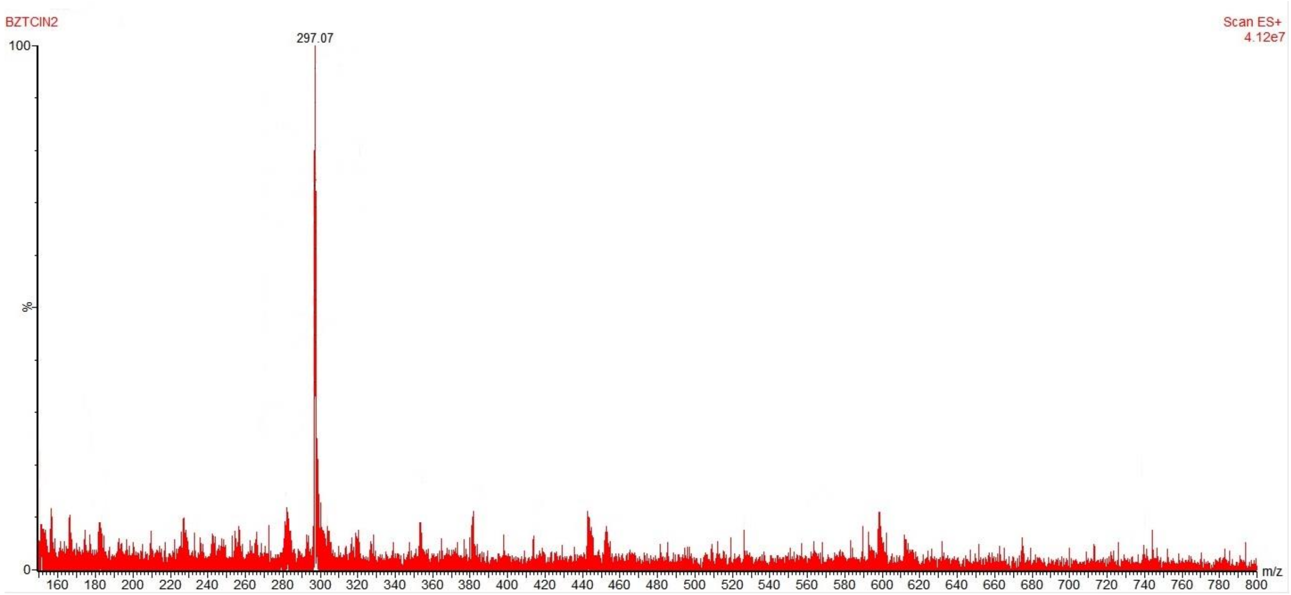

Figure S32. Mass spectrum of (E)-N-(benzo[d]thiazol-2-yl)-3-(4-hydroxyphenyl)acrylamide (**BZTcin2**).

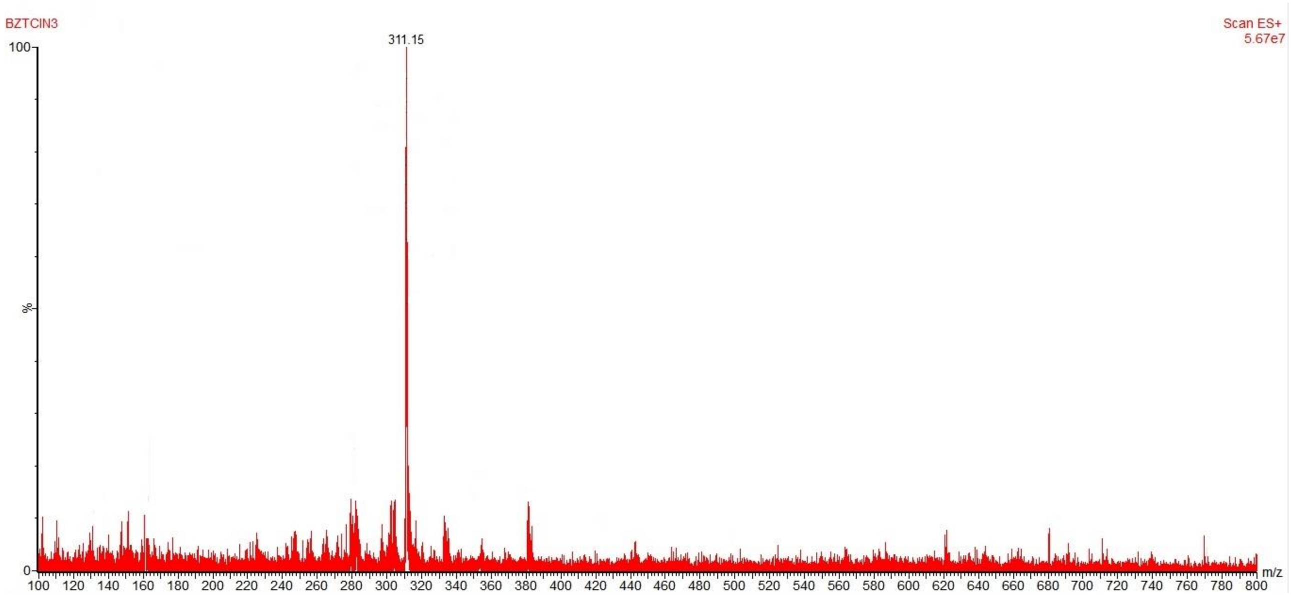

Figure S33. Mass spectrum of (E)-N-(benzo[d]thiazol-2-yl)-3-(4-methoxyphenyl)acrylamide (**BZTcin3**).

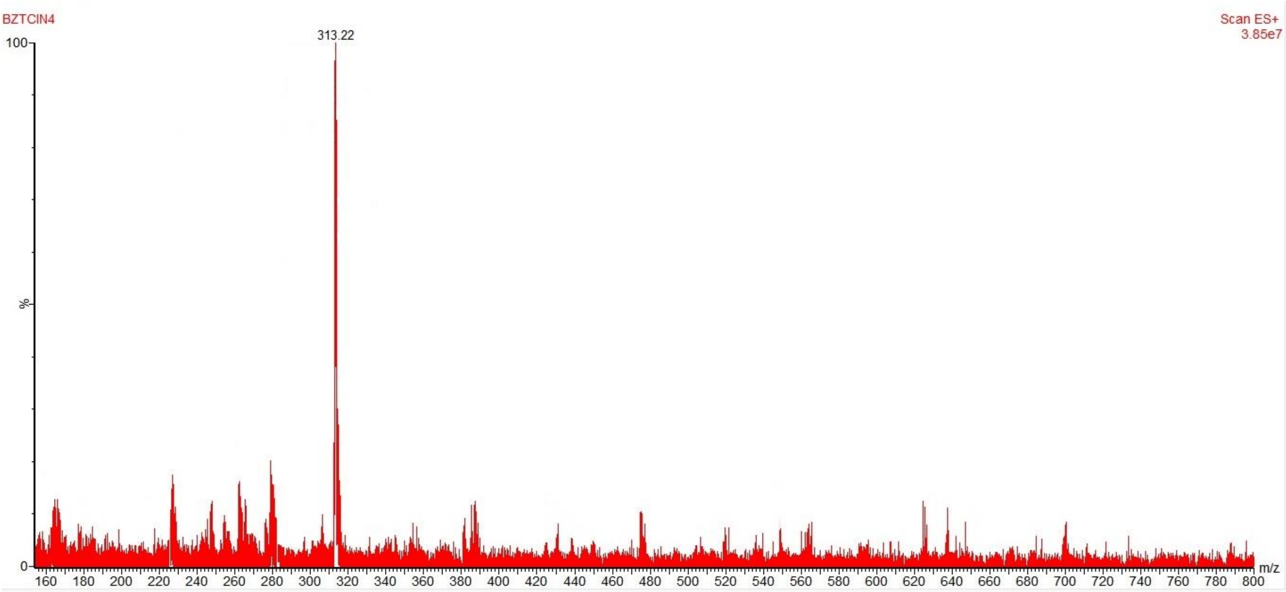

Figure S34. Mass spectrum of (E)-N-(benzo[d]thiazol-2-yl)-3-(3,4-dihydroxyphenyl)acrylamide (**BZTcin4**).

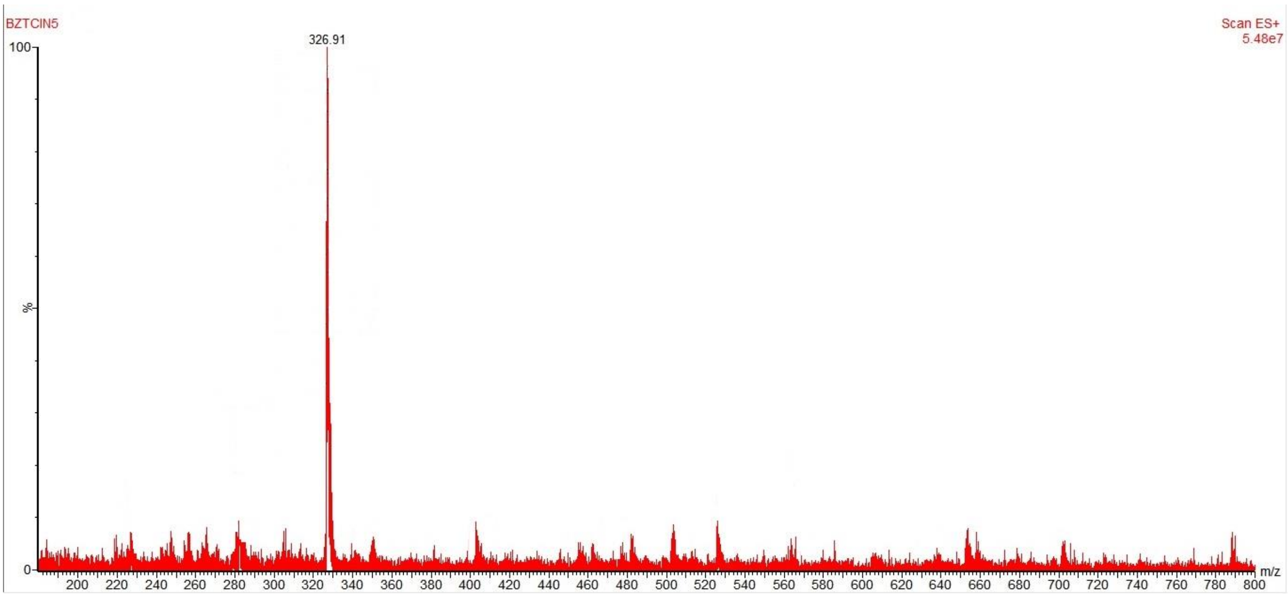

Figure S35. Mass spectrum of (E)-N-(benzo[d]thiazol-2-yl)-3-(3-hydroxy-4-methoxyphenyl)acrylamide (**BZTcin5**).
